# Investigating the role of NPR1 in dilated cardiomyopathy and its potential as a therapeutic target for glucocorticoid therapy

**DOI:** 10.3389/fphar.2023.1290253

**Published:** 2023-11-07

**Authors:** Yaomeng Huang, Tongxin Li, Shichao Gao, Shuyu Li, Xiaoran Zhu, Ying Li, Dangyang Liu, Weimin Li, Linquan Yang, Kunshen Liu, Zheng Zhang, Chao Liu

**Affiliations:** ^1^ Laboratory Center, The First Hospital of Hebei Medical University, Shijiazhuang, Hebei, China; ^2^ The First Cardiology Division, The First Hospital of Hebei Medical University, Shijiazhuang, Hebei, China; ^3^ Department of Cardiology, Tangshan Gongren Hospital, Tangshan, Hebei, China; ^4^ Department of Pharmacy, Hebei General Hospital, Shijiazhuang, Hebeie, China

**Keywords:** natriuretic peptide receptor 1, dilated cardiomyopathy, glucocorticoid, WGCNA, SsGSEA

## Abstract

**Background:** Dilated cardiomyopathy (DCM), a specific form of cardiomyopathy, frequently presents clinically with either left ventricular or biventricular enlargement, often leading to progressive heart failure. In recent years, the application of bioinformatics technology to scrutinize the onset, progression, and prognosis of DCM has emerged as a fervent area of interest among scholars globally.

**Methods:** In this study, core genes closely related to DCM were identified through bioinformatics analysis, including weighted gene co expression network analysis (WGCNA) and single sample gene set enrichment analysis (ssGSEA) and so on. The correlation was verified through experiments on DCM patients, DCM rat models, and core gene knockout mice. Subsequently, the effects of glucocorticoids on DCM and the regulation of core genes were observed.

**Result:** In the present study, natriuretic peptide receptor 1 (NPR1) was identified as a core gene associated with DCM through WGCNA and ssGSEA. Significant impairment of cardiac and renal function was observed in both DCM patients and rats, concomitant with a notable reduction in NPR1 expression. NPR1 KO mice displayed symptomatic manifestations of DCM, underscoring the pivotal role of NPR1 in its pathogenesis. Notably, glucocorticoid treatment led to substantial improvements in cardiac and renal function, accompanied by an upregulation of NPR1 expression.

**Discussion:** These findings highlight the critical involvement of NPR1 in the pathophysiology of DCM and its potential as a key target for glucocorticoid-based DCM therapy. The study provides a robust theoretical and experimental foundation for further investigations into DCM etiology and therapeutic strategies.

## 1 Introduction

Heart failure (HF) is a chronic disease caused by multiple complex factors and is primarily characterized by impaired systolic and/or diastolic function of the ventricles (Solomon et al., 2019; Tsutsui et al., 2021). Approximately 26 million individuals worldwide suffer from HF, and the number of deaths due to HF has reached 2 million annually, leading to an increasingly serious impact on public health (Benjamin et al., 2019; Salim et al., 2021).

HF is often caused by excessive cardiac load and primary myocardial injury, including myocardial diseases, ischemic myocardial injury, and myocardial metabolic disorders. Dilated cardiomyopathy (DCM) is one of the most common causes of HF (Petar et al., 2019). DCM, a type of cardiomyopathy, often manifests clinically as left ventricular or biventricular enlargement. The most common complications encountered in clinical practice include cardiac enlargement, progressive HF, arrhythmia, thromboembolism, and sudden cardiac death. During DCM progression, ventricular remodeling occurs in response to various factors and forms the basis for HF progression (Schultheiss et al., 2019).

DCM pathogenesis is highly complex and involves the interaction of multiple factors. Excessive cardiac load and primary myocardial injury are the main contributing factors. Genetic factors, infections, autoimmune responses, and long-term hypertension can lead to abnormal changes in the structure and function of the myocardium, ultimately resulting in DCM (Murtaza et al., 2019; Peters et al., 2020; Jain et al., 2021; Moeinafshar et al., 2021). The pathogenesis of DCM is related to ventricular remodeling, neurohumoral factors, inflammation, immune dysfunction, and abnormal molecular expression (Zhang et al., 2017; Murtaza et al., 2019; Havlenova et al., 2021). Multiple pathways and molecules are involved in this process; however, the detailed underlying mechanisms remain unclear. In recent years, with the development of high-throughput sequencing and gene chip technologies, the use of bioinformatics technology to explore the occurrence, development, and prognosis of diseases has become a hot topic for scholars worldwide (Hwang et al., 2018; Nayor et al., 2019; Rinschen et al., 2019; Sturm et al., 2019; Montaner et al., 2020).

The present study aimed to use bioinformatics technology to screen for DCM-related genes and investigate their mechanisms, with the purpose of revealing the pathogenesis of DCM and seeking treatment methods. The GSE3586 dataset, containing expression profiles related to DCM, was selected from the Gene Expression Omnibus (GEO) database. This study aimed to predict the core genes that may play crucial roles in disease progression at the molecular level through the enrichment of relevant molecular pathways associated with DCM. Furthermore, the phenotype of the core genes was validated to further support the results of the bioinformatics analysis through basic and clinical experiments. Additionally, the role of glucocorticoids in DCM treatment is discussed in this article with the purpose of providing a theoretical and experimental basis for exploring the pathogenesis of DCM and elucidating therapeutic methods. This study also provides a theoretical reference for the interpretation, early diagnosis, and treatment of DCM.

## 2 Materials and methods

### 2.1 Characteristics of the original data

The GSE3586 dataset was obtained from the GEO database, which is hosted by the National Center for Biotechnology Information (NCBI). The GSE3586 dataset includes gene expression profiles from 13 patients with DCM, tissue samples from the interventricular septum, and 15 normal controls without HF (Barth et al., 2006).

### 2.2 Processing of gene chip data and selection of differentially expressed genes (DEGs)

R software was used to perform separate principal component analysis (PCA) on the specimens from the two datasets to observe the distribution between the groups. The chip data were background-corrected and normalized using GEO2R software for each sample in the GSE3586 dataset. The criteria for selecting DEGs were set as |log2FC| > 0.2 and adj. *p* < 0.05. The names of the DEGs that appeared in both datasets were obtained. Volcano plots software for the DEGs obtained from the two datasets were generated using R software.

### 2.3 Weighted gene Co-expression network analysis (WGCNA)

The GSE3586 dataset consists of 15 samples from healthy controls and 13 samples from patients with DCM. The R package WGCNA in R version 4.3.2 was used to perform the WGCNA analysis. First, the “WGCNA” package was used to identify hub genes significantly associated with DCM. The expression profiles of the top 25% most variable genes in the cohort were selected as input, with a soft-thresholding power of *β* = 16 and a scale-free of R2 = 0.9. Next, the adjacency matrix was transformed into a topological overlap matrix (TOM). Modules were identified using hierarchical clustering (minimum module size = 100). The adjacency matrix was clustered and hub modules were determined. Pearson’s correlation coefficient between module membership (MM) and clinical trait congestive activity factor (CAF) score was calculated to select the strongest positive correlation for further analysis. The gene significance (GS) for each gene feature and MM in the central module was determined. Finally, genes within the modules were screened as potential DCM-related genes using thresholds of MM > 0.5 and GS > 0.8. CytoScape software, version 3.9.1, was used to identify HF-related pathways and extract core genes.

### 2.4 Construction of rat ischemic DCM model

As described in a previous study (Semb et al., 1998), the mice for 2 months, with a weight of approximately 200 g were selected to construct the ischemic DCM model 8 weeks after the left anterior descending coronary artery (left ventricular ejection fraction [LVEF%] <45%). Dexamethasone was selected as the representative drug for glucocorticoids in this study. Dexamethasone was administrated 8 weeks after surgery. This study unfolded in two principal stages. The initial phase featured a comparative experiment involving two groups: 6 healthy rats (CON group) and 6 rats with Dilated Cardiomyopathy (DCM group). The subsequent phase, comprised four healthy rats (CON) and 4 rats with DCM (DCM group). Within the latter group in this phase, DCM rats underwent a 12-h regimen of glucocorticoid treatment (DCM+GC group, Dexamethasone, intramuscular injection, 1 mg/kg) to assess the clinical efficacy of this particular glucocorticoid. The detailed experimental procedure is illustrated in the Supplementary Figure S1.

### 2.5 Echocardiographic assessment of rat cardiac function

Cardiac function of the rats 12 h after DEX administration was evaluated using a Vevo2100 Doppler ultrasound system as Supplementary Figure S1. Two-dimensional (2D) M-mode echocardiography was performed to measure heart rate, left ventricular end-diastolic diameter (LVEDD), left ventricular end-systolic diameter (LVESD), left ventricular end-diastolic volume (LVEDV), left ventricular end-systolic volume (LVESV), LVEF%, stroke volume (SV), left ventricular fractional shortening (FS%), and cardiac output (CO). Statistical analyses were performed on the collected data.

### 2.6 Masson’s trichrome staining

The mice for 2 months, with a weight of approximately 200 g were selected to construct the ischemic DCM model 8 weeks after the left anterior descending coronary artery. Heart tissues were extracted and sliced for Masson staining 12 h after administering DEX as Supplementary Figure S1. The detailed steps have been described previously (Zhou et al., 2022). Briefly, tissue sections were dewaxed and immersed in distilled water. Weigert’s iron hematoxylin staining solution was added for 3 min. After 15 s of differentiation using hydrochloric acid and alcohol, sections were rinsed with water. Masson’s blue staining solution was used to counterstain the sections until they exhibited a blue color, followed by rinsing with distilled water for 1 min. The sections were then stained with Ponceau red staining solution for 7–8 s and washed with a 0.2% weak acid solution for 1 min. They were then washed with a phosphomolybdic acid solution for 2 min and a 0.2% weak acid solution for 1 min. Aniline blue staining solution was added for 1.5 min, followed by washing with a 0.2% weak acid solution for 1 min. The sections were rapidly dehydrated with 95% ethanol for 2–3 s, followed by dehydration with absolute ethanol thrice for 5–10 s each time. The sections were then cleared thrice with xylene for 1–2 min each time before mounting. The degree of cardiac tissue fibrosis was evaluated by calculating the percentage of collagen positive blue area to total tissue area.

### 2.7 Western blotting

The detailed steps have been previously described (Taylor and Posch, 2014). Briefly, tissues were cut into small pieces and homogenized using a glass homogenizer. The myocardial membrane was purified using a Minute membrane separation kit (Inventory Biotechnologies, United States). The purity of the extracted membrane was assessed by reacting it with glyceraldehyde 3-phosphate dehydrogenase (GAPDH; Proteintech, Wuhan, China) and sodium–potassium adenosine triphosphatase (Na-K-ATP) antibodies (Abcam, United States, ab76020). The protein concentration was measured using a bicinchoninic acid (BCA) protein assay kit (Solarbio, Beijing, China). Total protein staining was performed using a Bio-Rad stain-free gel to confirm protein loading. The membrane containing the membrane proteins with 5 × loading buffer was denatured at 65°C for 10 min, separated on a 4%–10% standard Tris-glycine extended (TGX)-stain-free gel (Bio-Rad, Hercules, CA), and transferred to a polyvinylidene difluoride (PVDF) membrane (Millipore, Bedford, MA). The membrane was blocked with 5% skimmed milk at room temperature for 2 h, and then incubated with primary antibodies against NPR1 (Abcam, ab14356, diluted 1:1,000 and GeneTex, United States, GTX109810, diluted 1:1,000) and GAPDH (Proteintech, United States, Cat No. 10494-1-AP, diluted 1:10,000) at 4°C. The membrane was further incubated with goat anti-rabbit immunoglobulin G (IgG) H&L secondary antibody (Cat. no. ab205718, 1:1,000 dilution; Abcam, United States) at room temperature. The bands were visualized using an ultrasensitive electrochemiluminescence (ECL) kit (P0018AS, Beyotime) and an ECL imaging system (ChemiDoc XRS, Bio-Rad). The data were analyzed using ImageJ software (version 1.8.0, National Institutes of Health).

### 2.8 Single-sample gene set enrichment analysis (ssGSEA) for differential gene selection

To explore the biological events associated with the core gene natriuretic peptide receptor 1 (*Npr1*), the GSE181114 dataset from the GEO database was used. This dataset includes 944 HF samples, and the sequencing platform used was GPL30456 [HTA-2_0] Affymetrix Human Transcriptome Array 2.0 [GC_CHF_Blood_HTA_ENSG_Grch38]. Based on the expression levels of *Npr1*, the samples were divided into a high-expression group (top 25% of expression levels) and a low-expression group (bottom 25% of expression levels). ssGSEA was performed using GSEA 4.3.2 software (https://www.gseamsigdb.org/gsea/index.jsp) to evaluate the relevant molecular mechanisms and signaling pathways in patients with DCM. Gene sets with a nominal *p* < 0.05 and a false discovery rate (FDR) < 0.25 were considered statistically significant.

### 2.9 Generation of gene knockout (KO) mice

*Npr1* gene KO mice (NPR1^−/−^ mice) were designed and generated by Beijing Biostar Technologies Co., Ltd. based on previous experimental studies (Holtwick et al., 2002). In brief, specific guide RNAs (sgRNAs) targeting the 5′ and 3′ sites of the *Npr1* gene were designed, and a 4.0 kb chromosomal deletion (from exon 1) was introduced at the corresponding sites in the mouse genome. This resulted in the production of F0 generation mice, which were subjected to polymerase chain reaction (PCR) and sequencing for identification. F0 mice were bred with wild-type (WT) mice to obtain F1 generation heterozygous mice. F1 mice of both sexes were then selected to generate F2 mice of various genotypes. The genotypes of F2 mice were determined using genotyping. F2 mice with a complete NPR1 KO were selected and bred to expand the population.

### 2.10 Clinical blood sample and grouping

This study included blood samples from patients with decompensated DCM who were admitted to the Cardiology Department of the First Hospital of Hebei Medical University and healthy individuals of the same age group. This study was approved by the Medical Ethics Committee of our hospital. The inclusion criteria were as follows: 1) ejection fraction (EF) < 30%; 2) B-type natriuretic peptide (BNP) > 600 pg/mL or NT-proBNP >2000 pg/mL; 3) left LVED.d > 5.0 cm (females) and LVED.d > 5.5 cm (males), along with one of the following symptoms or signs: 1) jugular venous distension, ② chest X-ray indicating pulmonary congestion or the presence of moist rales at the bases of both lungs upon auscultation, ③ hepatomegaly or hepatic congestion, ④ or peripheral edema or signs of fluid retention. The exclusion criteria were as follows: ① hypertension; ② myocarditis, restrictive cardiomyopathy, or hypertrophic cardiomyopathy; ③congenital cardiomyopathy or alcoholic cardiomyopathy; ④ recent myocardial infarction, stroke, angina, or cardiac surgery within the past 3 months; ⑤ hemodialysis; ⑥ serum creatinine >3.0 mg/dL; and ⑦ systolic blood pressure >140 mmHg. This study was divided into two distinct phases. The initial phase encompassed a comparative analysis between two cohorts, comprising 11 healthy individuals (designated as the control [CON] group) and 12 patients diagnosed with Dilated Cardiomyopathy (DCM) (constituting the observation group). The subsequent phase involved a larger cohort, comprising 49 healthy individuals (CON) and 110 individuals diagnosed with DCM (observation group). Within the latter group, DCM patients underwent a 4-week regimen of glucocorticoid treatment (Prednisone, 1 mg/kg/day, with a maximum dose of 60 mg/day) to scrutinize the clinical efficacy of this intervention. After an 8-h fasting period, approximately 3 mL of peripheral venous blood was collected from the enrolled patients in the morning on an empty stomach and stored at −80°C in ethylenediaminetetraacetic acid (EDTA) anticoagulant tubes. There were no statistically significant differences in sex or age between the two groups (*p* > 0.05).

### 2.11 Whole blood PCR

Blood samples were transferred to RNA-free centrifuge tubes, and 1:3 red blood cell lysis buffer was added. The tubes were placed on ice for lysis, and the precipitate was collected. RNA was extracted from the precipitate using a standard RNA extraction method. Briefly, RNA was precipitated using phenol-chloroform-isopropanol, washed with 75% ethanol, air-dried, and dissolved in diethylpyrocarbonate (DEPC)-treated water, then its concentration was measured using a nanonucleic acid analyzer. The extracted RNA was used as a template for PCR. The primer sequences and amplification conditions were as follows:

Primer sequences:

NPR1:

Forward primer: 5′-AAG​AGC​CTG​ATA​ATC​CTG​AGT​ACT-3′

Reverse primer: 5′-TTG​CAG​GCT​GGG​TCC​TCA​TTG​TCA-3′

GAPDH:

Forward primer: 5′-TGG​GTG​TGA​ACC​ACG​AGA​A-3′

Reverse primer: 5′-GGC​ATG​GAC​TGT​GGT​CAT​GA-3′

The PCR protocol consisted of an initial denaturation at 95°C for 2 min (1 cycle), followed by denaturation at 95°C for 30 s, annealing at 55°C for 30 s, and extension at 72°C for 40 s (30–35 cycles). The PCR products were analyzed using agarose gel electrophoresis and a gel imaging system.

### 2.12 Statistical analysis

All statistical analyses were performed using SPSS software (IBM SPSS Statistics 23). Data are presented as mean ± standard deviation (SD). Normality and homogeneity of variance tests were conducted for all the data. For data that exhibited a normal distribution and homogeneity of variance, a one-way analysis of variance (ANOVA) was performed, followed by *post hoc* Least Significant Difference (LSD) comparisons. For data that exhibited a normal distribution but heterogeneity of variance, one-way ANOVA assuming equal variances was used. For data that did not follow a normal distribution or had heterogeneous variances among the groups, a parametric Kruskal–Wallis test was used for comparisons between groups. Statistical significance was set at *p* < 0.05.

## 3 Results

### 3.1 Experimental flowchart

**FIGURE 1:**
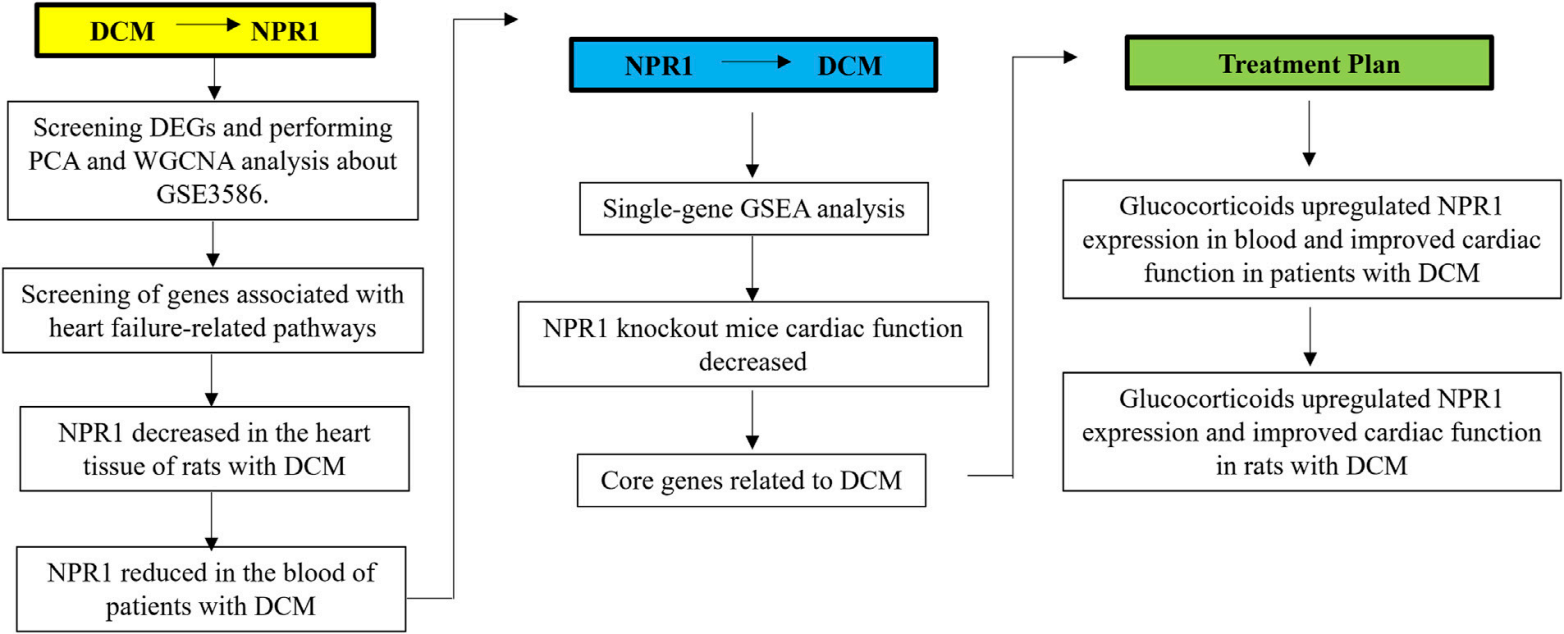
Experimental flowchart.

A detailed flowchart of this process is presented in Figure 1. This study was based on the GSE3586 dataset from GEO to explore the differentially expressed genes and core genes related to the disease in patients with DCM using WGCNA. The above WGCNA results were verified through basic experiments in DCM rats and patients with DCM.

Subsequently, ssGSEA was used to investigate the pathways related to the core gene *Npr1* and found the existence of two pathways related to DCM, which confirmed reversely the close correlation between *Npr1* gene and DCM. It was confirmed through NPR1 knockout mice that systemic knockout of NPR1 gene can cause DCM model.

Additionally, it was found that glucocorticoids can significantly improve DCM rats and patients with DCM, and significantly reversed the downward trend of NPR1 in the DCM model.

### 3.2 PCA and WGCNA analysis associated with GSE3586

**FIGURE 2:**
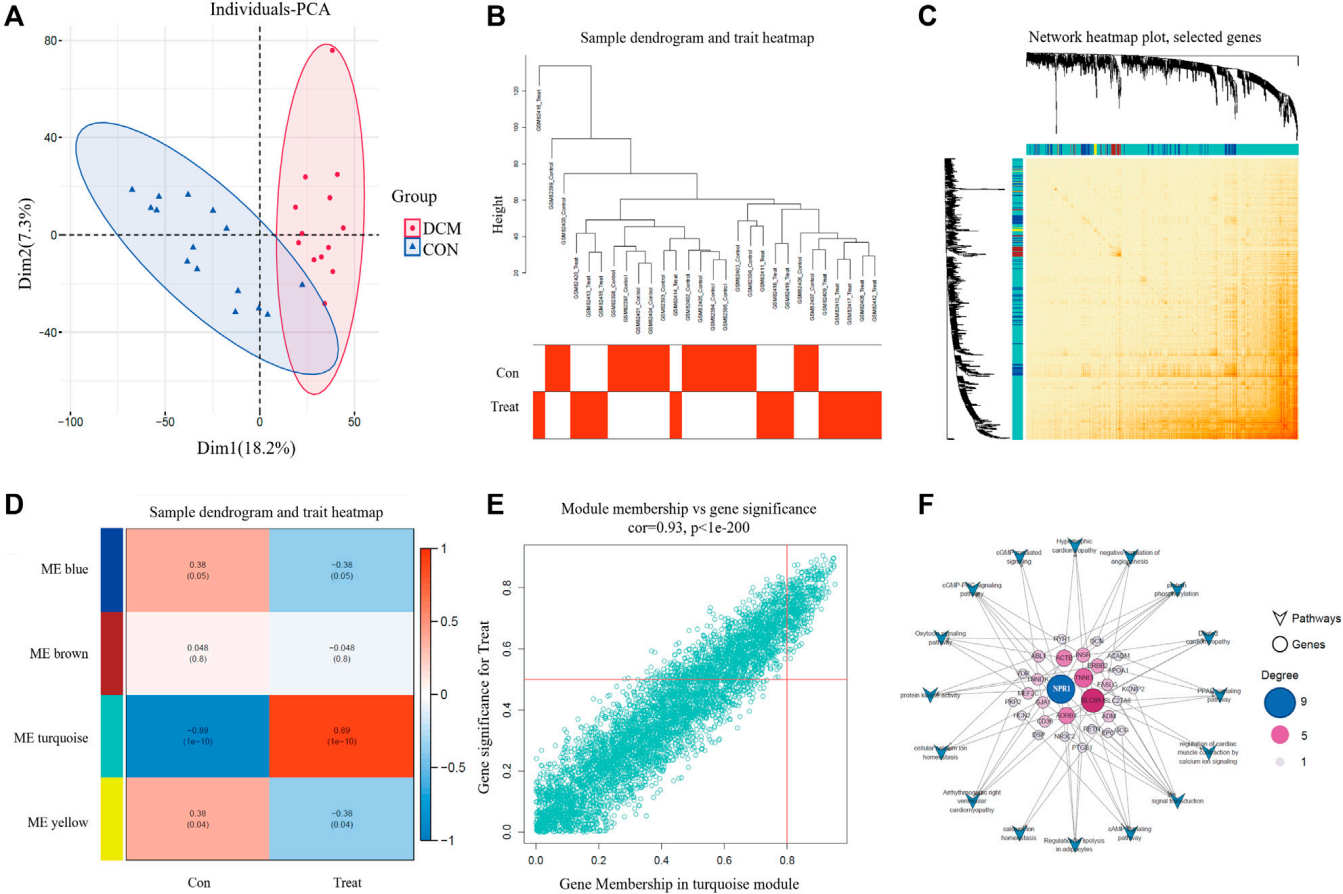
PCA and WGCNA analysis. **(A)** PCA diagram of CON and DCM groups; **(B)** Tree view and heat map; **(C)** Network heatmap; **(D)** module-related analysis and scoring; **(E)** Modules with significant correlation between MM and GS, and key genes related to DCM; **(F)** The relationship between different genes and DCM related pathways.

PCA was used to visualize the scatter plot of the GSE3586 chip data. Each point in the scatter plot represents a sample. The clustering results for the normal and DCM groups in both datasets showed consistent clustering (Figure 2A). Co-expression modules were constructed using WGCNA based on publicly available data (GSE3586). Pearson’s correlation coefficient was used to cluster the samples and obtain a sample-clustering tree. A soft threshold of 16 (based on the scale-free topology criterion with R^2 = 0.9) was used to create a scale-free network. The adjacency matrix was then transformed into a TOM (Figures 2B, C) to display the similarity between nodes by considering weighted correlations. To explore the relationship between these modules and the clinical data, we analyzed the GSE3586 dataset and identified four modules. Among these four modules, the turquoise module exhibited a high correlation with DCM scores (cor = 0.89, P = 1e-10) (Figure 2D). The module-trait relationships of the turquoise module exhibited a significant correlation. We further identified 375 key genes related to DCM (Figure 2E).

Kyoto Encyclopedia of Genes and Genomes (KEGG) pathway analysis of the 375 key genes revealed several pathways related to HF, including arrhythmogenic right ventricular cardiomyopathy, regulation of lipolysis in adipocytes, the peroxisome proliferator-activated receptor (PPAR) signaling pathway, the cyclic guanosine monophosphate-protein kinase G (cGMP-PKG) signaling pathway, DCM, signal transduction, regulation of cardiac muscle contraction by calcium ion signaling, the cyclic adenosine 3,5-monophosphate (cAMP) signaling pathway, cellular calcium ion homeostasis, protein phosphorylation, the oxytocin signaling pathway, negative regulation of angiogenesis, hypertrophic cardiomyopathy, protein kinase activity, and calcium ion homeostasis. By intersecting the DEGs with these pathways, we determined that NPR1was associated with nine pathways, ranking first in terms of the number of pathways involved (Figure 2F). This preliminary evidence suggests that the NPR1 gene is closely related to DCM.

### 3.3 Expression of NPR1 in rat heart tissue decreased significantly in ischemic DCM model rats

**FIGURE 3:**
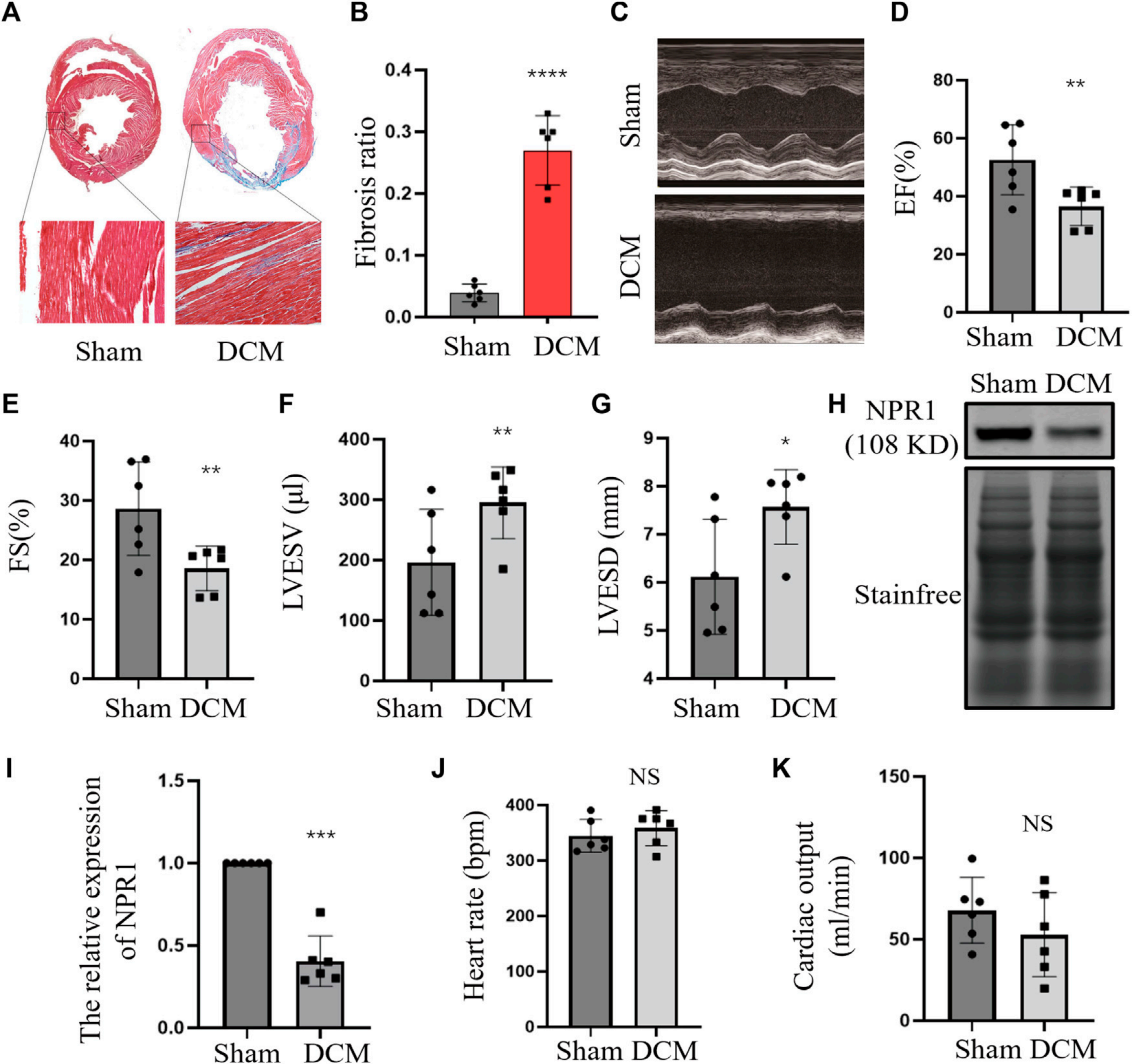
Downregulation of NPR1 expression in DCM rats. **(A)** Rat heart tissues with Masson’s trichrome staining; **(B)** Rat heart fibrosis analysis; **(C–G)** Rat heart echocardiography and EF%, FS%, LVESV, and LVESD analysis; **(H–I)** NPR1 expression and analysis. **(J)** Rat heart rate; **(K)** Rat cardiac output; Data are presented as mean ± SD (CON, *n* = 6; DCM, *n* = 6). *p < 0.05, **p < 0.01, ***p < 0.001 vs. Sham group. Abbreviations: EF, ejection fraction; FS, fractional shortening; LVESV, left ventricular end-systolic volume; LVESD, left ventricular end-systolic diameter. NS, no significance.

Masson’s trichrome staining was performed to evaluate the degree of cardiac fibrosis. Compared with the sham group, the ischemic DCM group exhibited a substantial increase in the degree of myocardial fibrosis (Figures 3A, B). Echocardiography results indicated that, compared with the sham group, the DCM group exhibited a significant decrease in EF% and fractional shortening (FS%), an obvious increase in LVESV and LVESD were considerably increased (Figures 3C–G), and no significant changes in heart rate and cardiac output (Figures 3J, K). Moreover, there was a notable decrease in NPR1 expression in the cardiac tissue membrane (Figures 3H, I). The purity of the extracted membrane was assessed through reaction with GAPDH and Na-K-ATP antibodies (Supplementary Figure S2). In addition, biochemical results showed that, compared with the sham group, the DCM group exhibited a decrease in urine volume, urinary sodium, urinary creatinine, and creatinine clearance (Supplementary Figures S3A–D).

### 3.4 Changes in whole blood indices of patients with DCM

**FIGURE 4:**
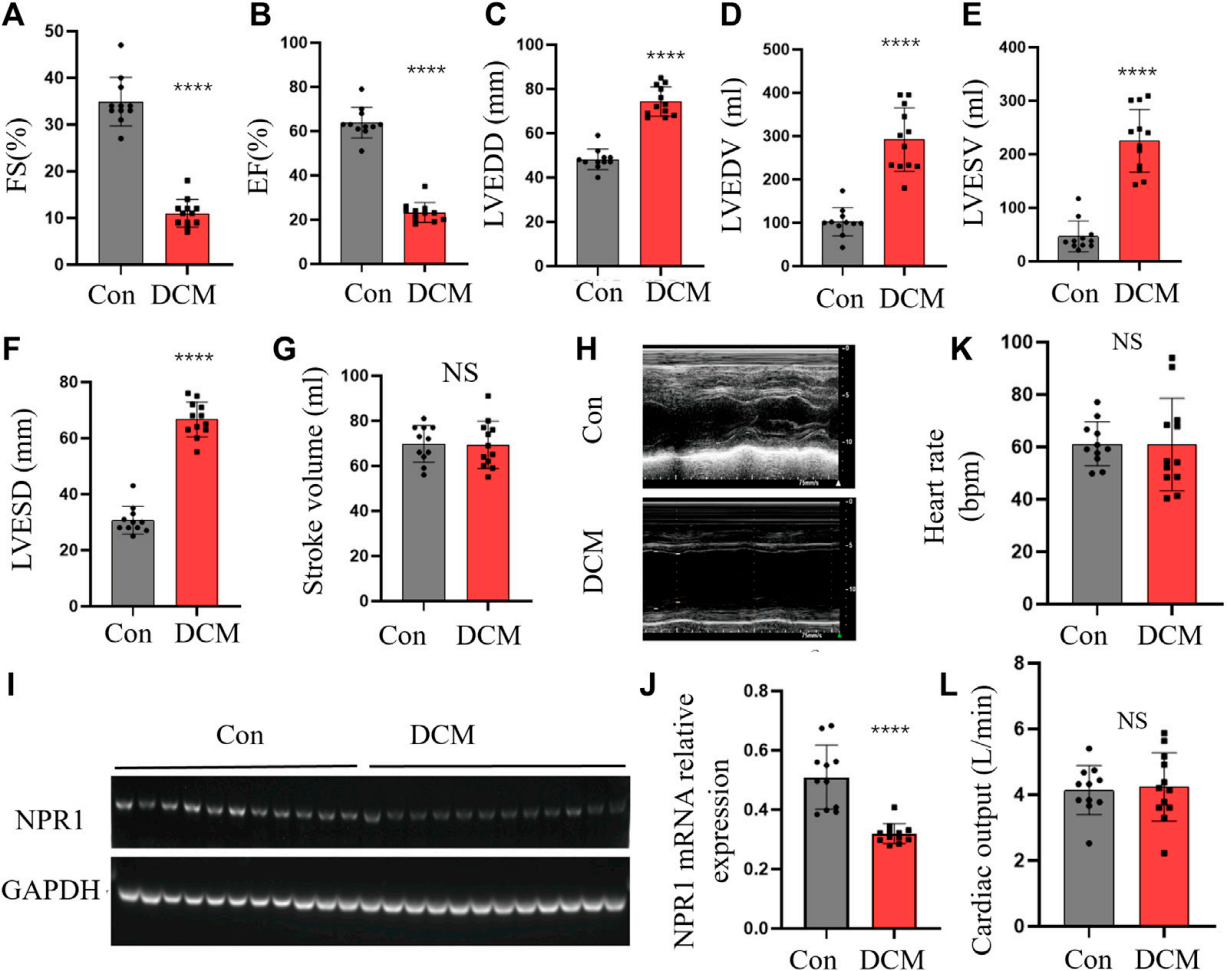
Changes in cardiac function indicators. **(A–G)** FS, EF, LVEDD, LVEDV, LVESV, EF, LVESD, and SV in different groups; **(H)** Echocardiography in different groups; **(I–J)** NPR1 expression and analysis. **(K)** Heart rate in different groups; **(L)** Cardiac output in different groups; Data are presented as mean ± SD (CON, *n* = 11; DCM, *n* = 12). ****p < 0.0001 vs. Con group. Abbreviations: LVEDD, left ventricular end-diastolic diameter; EF, ejection fraction; LVEDV, left ventricular end-diastolic volume; LVESV, left ventricular end-systolic volume; FS, fractional shortening; LVESD, left ventricular end-systolic diameter; CREA, creatinine; UREA, urea; eGFR, estimated glomerular filtration rate. NS, no significance.

A total of 11 patients with DCM and 12 healthy control subjects (CON group) were included, with no statistical differences in sex, age, smoking status, presence of renal disease, or diabetes (Supplementary Table S1). Compared with the CON group, the DCM group met the following criteria for cardiac functional indices: a certain decrease in EF% and FS% (Figures 4A, B); a noticeable increase in LVEDD, LVEDV, LVESV, and LVESD (Figures 4C–F); no definite change in SV, heart rate and cardiac output (Figures 4H, K, L). In addition, the expression of the NPR1 mRNA in whole blood was significantly decreased (Figures 4L, M). At this time, the DCM group exhibited a significant increase in blood creatinine and blood urea levels and a dominant decrease in the glomerular filtration rate (GFR) (Supplementary Figures S4A–C).

### 3.5 ssGSEA

**FIGURE 5:**
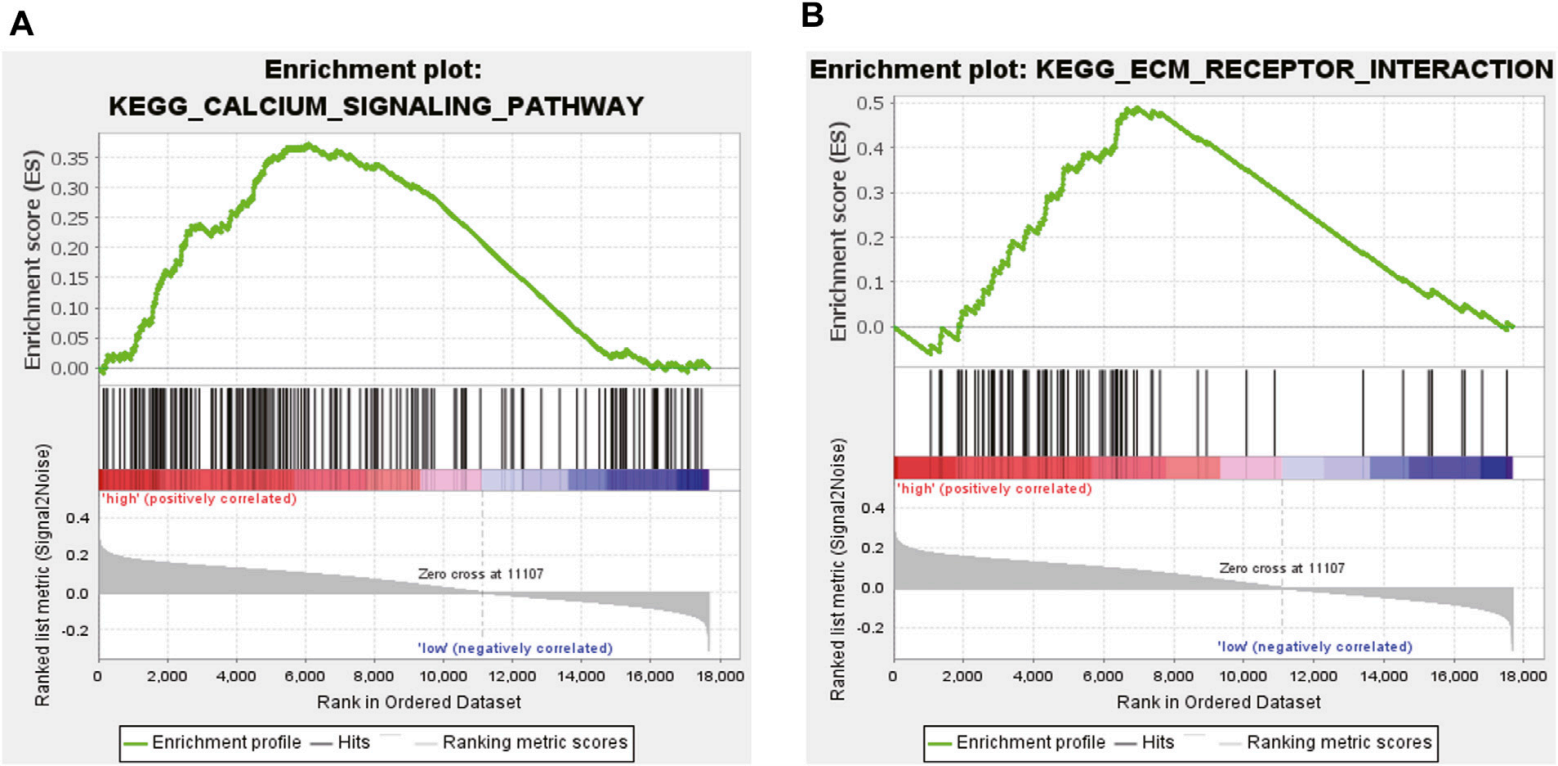
ssGSEA analysis. **(A)** Calcium signaling pathway; **(B)** ECM receptor interaction pathway.

After excluding ten Gene Ontology (GO) terms from the 186 genes, the remaining 176 GO terms were used for subsequent analyses. We determined that 105 gene terms were upregulated and nine gene terms were significantly enriched at nominal *p*-value (NOM *p*) < 0.05, normalized enrichment score (NES) > 1.0, and false discovery rate (FDR q) > 0.25. Two of the enriched gene terms were related to DCM, namely, “Calcium signaling Pathway” and “Extracellular Matrix (ECM) Receptor Interaction.” A snapshot of the enrichment results is shown in Figures 5A, B.

### 3.6 NPR1 KO impaired cardiac function in mice

**FIGURE 6:**
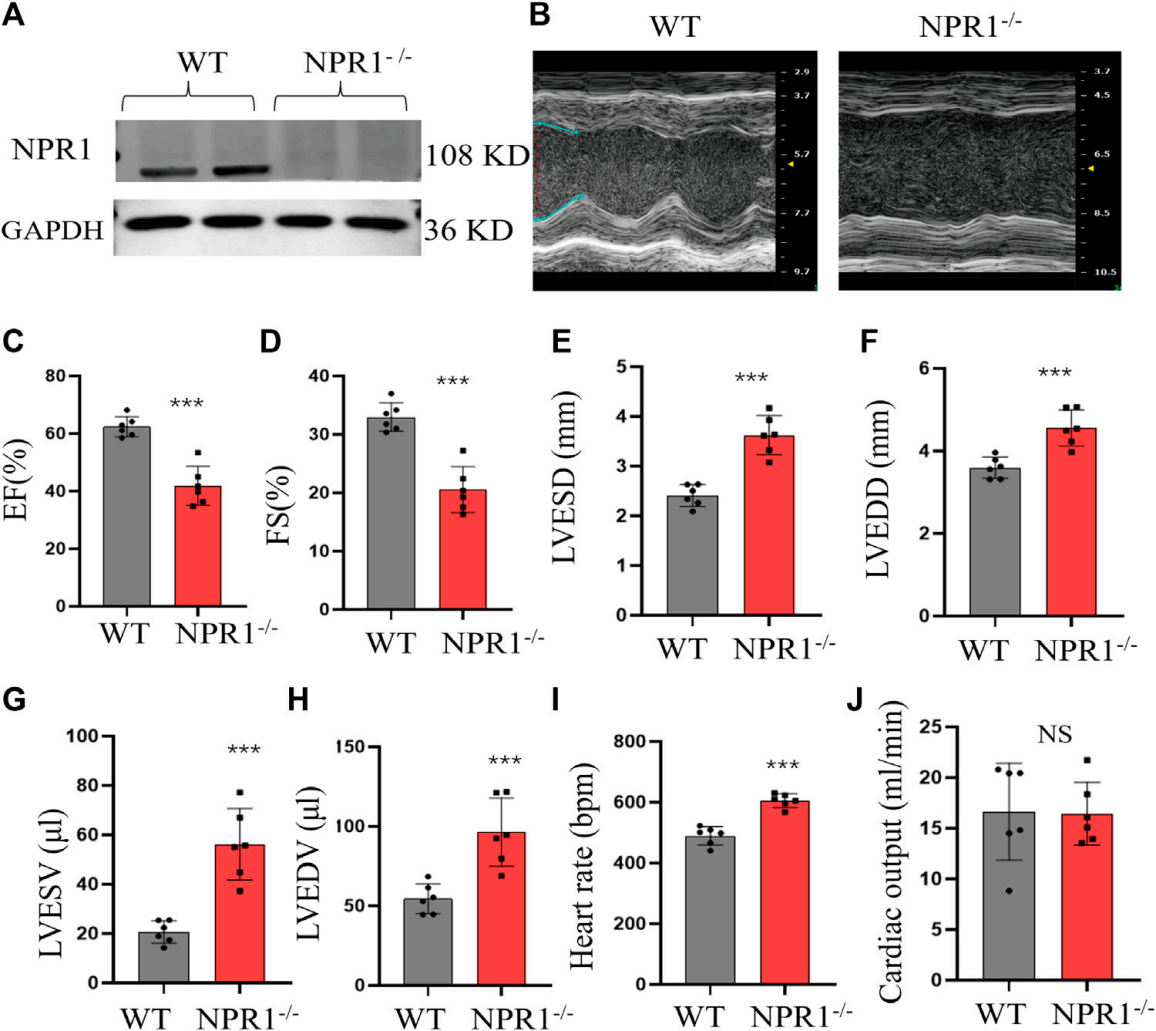
Change in cardiac function indicators in NPR1 KO mice. **(A)** NPR1 expression in mouse heart tissues from different groups; **(B)** Echocardiograms of mice in each group; **(C–H)** EF, FS, LVESD, LVEDD, LVESV, and LVEDV in different groups. **(I–J)** Heart rate and cardiac output in different groups. Data are presented as mean ± SD (WT *n* = 6, NPR1^−/−^ *n* = 6). *p < 0.05, ***p < 0.001 vs. WT group. Abbreviations: EF, ejection fraction; LVESD, left ventricular end-systolic diameter; LVEDD, left ventricular end-diastolic diameter; LVESV, left ventricular end-systolic volume; LVEDV, left ventricular end-diastolic volume; and FS, fractional shortening. NS, no significance.

The cardiac tissue of NPR1^−/−^ mice showed a lack of expression of NPR1 (Figure 6A). Compared with WT mice at 7 weeks of age, NPR1-ko mice exhibited a marked decrease in EF% and FS% (Figures 6B–D), a conspicuous increase in diastolic and systolic volumes and diameters (Figures 6E–H), and no significant change in heart rate and cardiac output (Figures 6I, J). Furthermore, biochemical results showed that, compared to WT mice, NPR1^−/−^ mice showed a decrease in urine volume, urinary sodium, urinary creatinine, creatinine clearance and blood creatinine concentration (Supplementary Figures S5A–E). In addition, the results indicate that compared with WT mice, NPR1 KO mice showed a significant increase in systolic blood pressure (SBP), diastolic blood pressure (DBP), and mean arterial pressure (MBP). After DEX treatment, the above indicators showed a significant decrease and tended to normal (Supplementary Figures S6A–C).

### 3.7 Glucocorticoids improved cardiac and renal function in the DCM group and upregulated NPR1 mRNA expression

**FIGURE 7:**
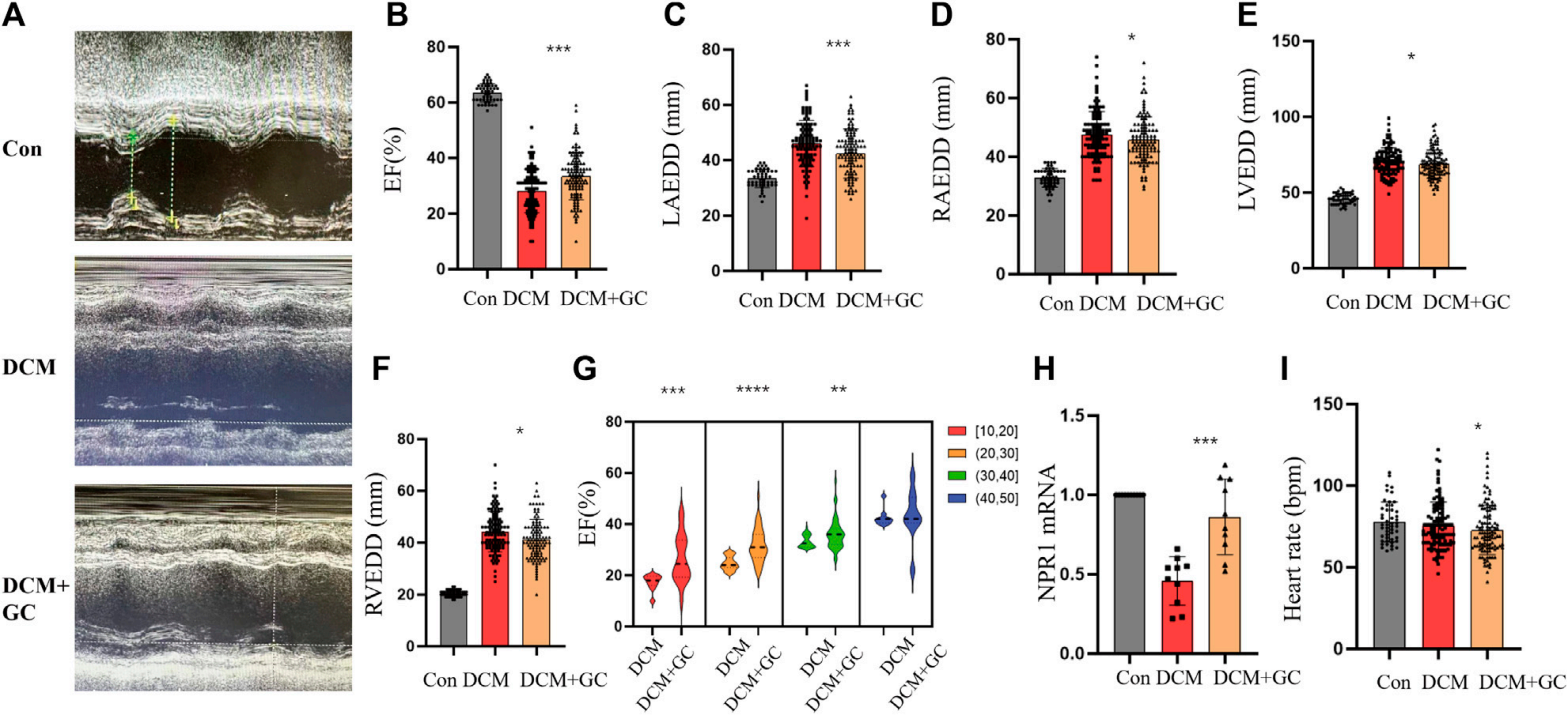
Glucocorticoids improve cardiac function indicators in patients with DCM. **(A)** Echocardiograms in different groups; **(B–F)** Changes in EF, LAEDD, RAEDD, LVEDD and RVEDD in different groups. Data are presented as mean ± SD (CON, *n* = 49; DCM, *n* = 110; and DEX+GC, *n* = 110); **(G)** In different ranges of 10–20, 20–30, and 30–40, DEX affects EF. ([10–20] *n* = 20, [20,30] *n* = 39, [30,40] *n* = 44, [40,50] *n* = 7); **(H)** Changes in NPR1 mRNA expression were observed in different groups. **(I)** Heart rate in different groups. Data are presented as mean ± SD (CON, *n* = 10; DCM, *n* = 10; and DEX+GC, *n* = 10) *p < 0.05, **p < 0.01, ***p < 0.001 vs. DCM group. Abbreviations: EF, ejection fraction; RVEDD, right ventricular end-diastolic diameter; NS, no significance.

There were no statistically significant differences in sex, age, coronary heart disease, hypertension, hyperlipidemia, atrial fibrillation or flutter, valvular disease, peripheral vascular disease, percutaneous coronary intervention (PCI), coronary artery bypass graft surgery (CABG), transient ischemic attack (TIA), chronic obstructive pulmonary disease (COPD), chronic kidney disease (CKD), thyroid dysfunction, acute myocardial infarction (AMI), abnormal liver function, or anemia (Supplementary Table S2). Compared with the CON group, the DCM group exhibited a notable decrease in the EF and a significant decrease in NPR1 mRNA levels in whole blood (Figures 7A, B, H). The left atrium, right atrium, left ventricle, and right ventricle exhibited demonstrable dilation (Figures 7C–F). After glucocorticoid treatment, patients with DCM showed a notable increase in NPR1 mRNA levels in whole blood (Figure 7H), and the enlarged left atrium, right atrium, left ventricle, and right ventricle all exhibited visible reduction (Figures 7C–F), accompanied by a significant recovery in the EF (Figure 7B). Further stratified analysis of EF revealed that when the EF was in the ranges 10–20, 20–30, 30–40, and 40–50, glucocorticoid treatment resulted in a distinct recovery of the EF (*p* < 0.05). However, when the EF was in the range 40–50, there was no significant difference in the regulation of the EF by glucocorticoids (Figure 7G). Besides, glucocorticoids can reduce heart rate in DCM patients (Figure 7I). Furthermore, these biochemical results revealed that, compared with the CON group, patients with DCM exhibited a visible increase in urea creatinine, and uric acid levels, which substantially decreased after glucocorticoid administration (Supplementary Figures S7A, B).

### 3.8 Glucocorticoids improved cardiac and renal function in DCM rats and upregulated NPR1 expression

**FIGURE 8:**
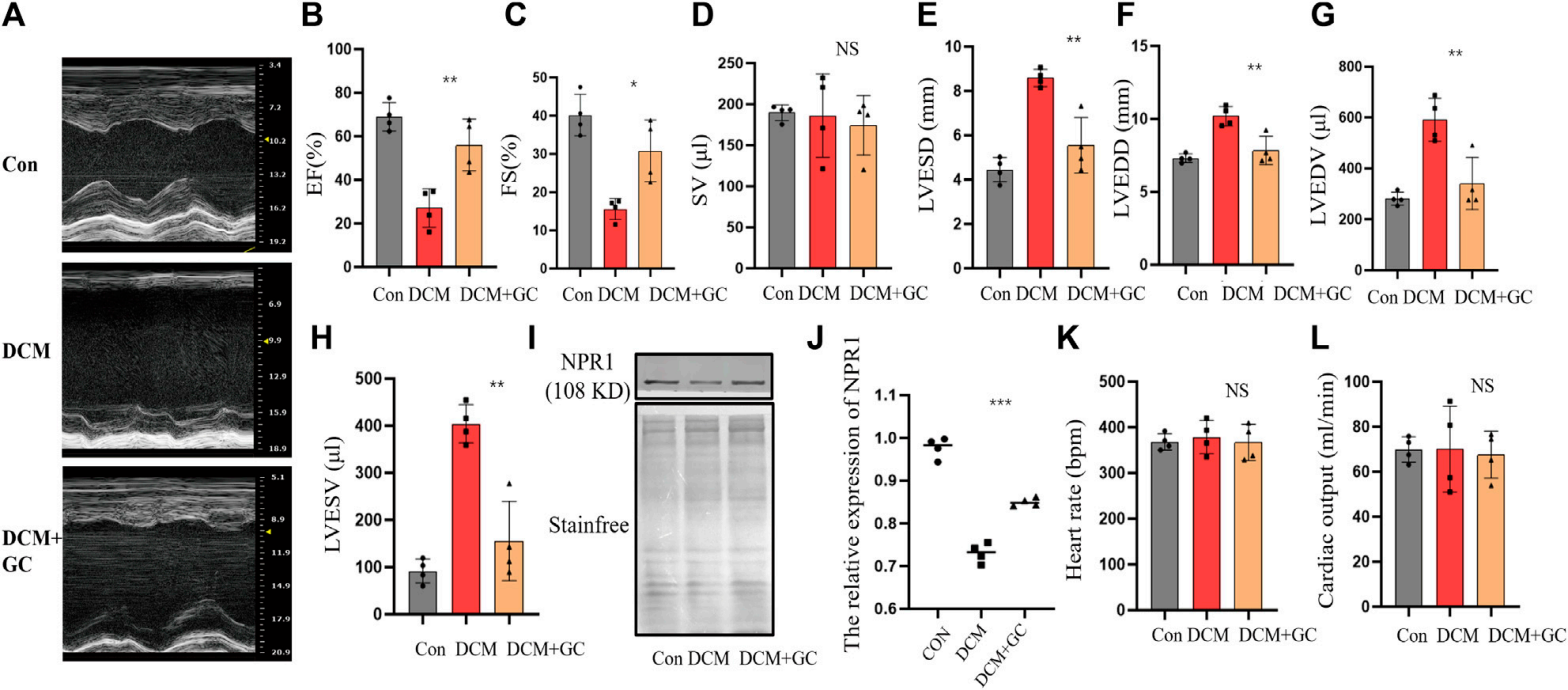
Glucocorticoids improve cardiac function indicators in rats with DCM. **(A)** Echocardiograms in different groups; **(B–H)** Changes in EF, FS, SV, LVESD, LVEDD, LVEDV, and LVESV in the echocardiography of each group. **(I–J)** Changes in NPR1 protein expression in different groups. **(K–L)** Heart rate and cardiac output in different groups. Data are presented as mean ± SD (CON, *n* = 4; DCM, *n* = 4; and DEX+GC, *n* = 4). *p < 0.05, ***p < 0.001, ***p < 0.0001 vs. DCM group. Abbreviations: EF, ejection fraction; FS, fractional shortening; LVESD, left ventricular end-systolic diameter; LVEDV, left ventricular end-diastolic volume; LVEDD, left ventricular end-diastolic Diameter; LVESV, left ventricular end-systolic volume; NS, no significance.

Compared to the CON group, rats with DCM exhibited a significant decrease in EF and FS and an emphatic increase in LVESD, LVEDV, LVEDD, and LVESV (Figures 8A–C, E–H). After glucocorticoid treatment, the EF and FS significantly increased, whereas LVESD, LVEDD, LVEDV, and LVESV dramatically decreased. After glucocorticoid treatment, DCM rats exhibited a conspicuous increase in NPR1 mRNA levels in whole blood (Figures 8I, J) and no significant change in heart rate and cardiac output (Figures 8K, L). In addition, there was no statistically significant difference in SV between the groups (Figure 8D).

Biochemical results showed that compared with the CON group, patients with DCM experienced significant fluctuations in urinary sodium, creatinine, and creatinine clearance, which significantly increased after glucocorticoid administration (Supplementary Figures S8A–C).

## 4 Discussion

DCM is a type of cardiomyopathy characterized by cardiac enlargement, progressive HF, arrhythmia, thromboembolism, and sudden cardiac death (Schultheiss et al., 2019). The incidence and mortality rates of DCM are increasing annually, particularly among younger individuals. Patients with DCM who progress to HF generally have a poorer prognosis than those without DCM. However, the molecular mechanisms underlying the pathophysiology of DCM remain to be fully understood, which highlights the importance of exploring the susceptibility modules and genes associated with DCM.

In the present study, we used published data from the GSE3586 dataset to construct co-expression modules using WGCNA. To the best of our knowledge, this is the first study to explore the susceptibility modules and genes associated with DCM using WGCNA. By thoroughly reanalyzing the GSE3586 dataset, we obtained four modules, among which the turquoise module showed a high correlation with DCM scores (cor = 0.89, P = 1e-10). The turquoise module exhibited significant correlations between MM and GS, allowing the identification of 375 key genes associated with DCM.

To gain further insight into the molecular pathways involved in DCM, we performed a KEGG analysis of the 375 key genes. The analysis revealed several pathways predominantly associated with HF, including arrhythmogenic right ventricular cardiomyopathy, regulation of lipolysis in adipocytes, PPAR signaling pathway, cGMP-PKG signaling pathway, DCM, signal transduction, regulation of cardiac muscle contraction by calcium ion signaling, cAMP signaling, cellular calcium ion homeostasis, protein phosphorylation, the oxytocin signaling pathway, negative regulation of angiogenesis, hypertrophic cardiomyopathy, protein kinase activity, and calcium ion homeostasis. By intersecting the differential genes with the aforementioned pathways and arranging them in descending order of the number of DEGs involved in each regulatory pathway, NPR1 was ranked first with its association with nine pathways. This preliminary evidence suggests a close correlation between the NPR1 and DCM.

Basic experiments were conducted to confirm these results. First, an ischemic DCM rat model was established by ligating the left anterior descending coronary artery. Rats with a noticeable decrease in EF% and FS% and a significant increase in LVEDD and LVESV (*p* < 0.05) exhibited characteristics consistent with DCM. The expression of NPR-A in the cardiac membrane was decreased. To further investigate the relationship between NPR1 and DCM *in vitro*, 11 healthy individuals and 12 patients diagnosed with DCM were included, while considering factors such as sex, age, smoking, and chronic diseases were considered. Patients with DCM showed a dominant decrease in EF% and FS%, and a significant increase in LVEDD, LVESD, LVEDV, and LVESV, meeting the criteria for DCM. The expression of NPR1 mRNA in whole blood, as determined using PCR, was significantly lower in patients with DCM than in healthy individuals (*p* < 0.05), indicating that DCM status can reduce NPR1 expression. To further elucidate the correlation between NPR1 and the DCM model, NPR1 KO mice were generated. Compared with WT mice, NPR1^−/−^ mice exhibited a significant decrease in EF% and FS% and a marked increase in diastolic and systolic volumes and diameters, further confirming the role of NPR1 deficiency in promoting the occurrence and development of DCM.

Based on bioinformatics analysis and WGCNA, we analyzed DEGs in 13 heart muscle samples from patients with DCM and 15 donor heart muscle samples from patients without DCM obtained from the GSE3586 dataset. Among the DEGs in DCM, the NPR1 was closely associated with DCM as a core gene. The GSE181114 dataset was used to explore the biological events associated with the NPR1. ssGSEA confirmed the high expression of the *Npr1* gene and identified two pathways closely related to DCM, namely, “Calcium Signaling Pathway” and “ECM Receptor Interaction.” These findings provide further evidence for the significant impact of NPR1 on DCM. Bioinformatics analysis validated the close correlation between NPR1 and DCM from both positive and negative aspects.

Regarding the NPR1 KO strategy, various methods are currently available, with insertion- and splicing-type deletions being the most commonly used (Lopez et al., 1995; Knowles et al., 2001; Taylor and Posch, 2014; Gogulamudi et al., 2019). In this study, we selected a splicing-type deletion. However, in contrast to our KO strategy, several experimental findings have suggested that NPR1 KO mice primarily exhibit renal fibrosis, increased inflammation, and decreased function (Gogulamudi et al., 2019). Left ventricular dilation and cardiac dysfunction are more commonly observed after cardiac load-induced heart injuries, such as transverse aortic constriction (TAC), than under basal conditions (Knowles et al., 2001). Another study found that at 12 weeks of age, NPR1^−/−^ mice exhibited worsening cardiac function, characterized by hypertension-related left ventricular hypertrophy and impaired cardiac systolic and diastolic functions. The presence of DCM and direct indicators of cardiac function such as EF, FS, and LVEDD were not considered (Lopez et al., 1995). Therefore, further investigations are required to determine whether NPR1 deficiency leads to DCM and HF.

Previous studies using the same KO strategy did not show definite differences in EF and FS between *Npr1* global KO mice and WT mice, although indices of myocardial hypertrophy, such as end-diastolic interventricular septum, posterior wall, heart weight-to-body weight ratio (HW/BW), left ventricular weight-to-body weight ratio (LVW/BW), and right ventricular weight-to-body weight ratio (RVW/BW) were upregulated (Kilic et al., 2005). One hypothesis is that although the same gene KO strategy was applied in both experiments, slight differences may exist in the actual cleavage sites after sgRNA cleavage. Genes that directly modulate protein expression or indirectly regulate gene transcription to affect the cardiac contractile function may exhibit differences.

Nakagawa et al. (2014) conducted experiments using the same KO strategy and determined that the baseline levels of weight-to-tibia length, LV longitudinal cardiomyocyte area, wet lung weight/TL, and LV interstitial collagen fractions in heart-specific NPR1 KO mice were not significantly different. However, when subjected to TAC, the KO group exhibited a certain degree of increase in weight-to-tibia length, LV longitudinal cardiomyocyte areas, wet lung weight/TL, LV interstitial collagen fractions, and myocardial fibrosis levels compared to the CON group, although statistical significance was not explicitly mentioned in the paper. Based on this, it can be inferred that the specific KO of cardiac NPR1 using the method employed in this study increases the susceptibility to HF but does not result in DCM-related HF manifestations. When subjected to TAC, the HF phenotype was significantly activated, exhibiting significant changes consistent with previous research (Lopez et al., 1995).

Our research mainly focused on DCM caused by the overall deletion of the *Npr1* gene without additional surgical intervention, such as ligation, which can be partly understood as the increased susceptibility to DCM-related HF caused by the deletion of the cardiac *Npr1* gene. Knocking out the NPR1 from other tissues causes significant damage to organs such as the kidneys and can serve as a stimulating factor to induce DCM-related HF. The advantage of this model is that it does not require the cumbersome steps of TAC surgery followed by myocardial-specific NPR1 KO; instead, direct systemic KO results in phenotypic changes associated with DCM-related HF.

In conclusion, our study used WGCNA, bioinformatics analysis, and experimental validation to reveal a close correlation between the NPR1 and DCM-related HF. Additionally, it would be valuable to further investigate the effects of the NPR1 deficiency on the development of DCM and HF using different KO strategies and animal models. Exploring the roles of the other genes and molecular pathways identified in this study may also contribute to a more comprehensive understanding of the pathogenesis of DCM.

Glucocorticoids (GC) were historically employed in heart failure (HF) treatment. In the 1950s, Camara et al. demonstrated remarkable efficacy with low-dose GC, suggesting a potential link to HF-induced adrenal cortical ischemia and subsequent GC deficiency (Camara and Schemm, 1955). Other studies supported GC’s alleviation of edema and fatigue in Addison’s patients (Mickerson and Swale, 1959; Teodorescu et al., 1959). Some scholars proposed that GC, akin to mineralocorticoids, led to sodium and water retention, possibly exacerbating cardiac preload in HF patients (Massari et al., 2012). The long-term use of GC can result in distinctive side effects, such as “full moon face” attributed to protein degradation and fat redistribution, rather than sodium-water retention. Notably, the sodium-retaining effect of GC lacks robust scientific and clinical backing. Research underscores GC’s capacity for potent diuresis and symptomatic relief in refractory HF cases with fluid retention, without adverse impacts on other organ functions (Liu C et al., 2006; Liu C et al., 2007). Fundamental studies have demonstrated that GC can inhibit the renin-angiotensin-aldosterone system (RAAS), activate the natriuretic peptide (NP) and nitric oxide (NO) systems, enhance renal blood flow, ameliorate kidney function, and exert robust diuretic effects (Liu C et al., 2010; Liu C et al., 2016). Experiments on HF rats have revealed that GC, through the inhibition of the arginine vasopressin (AVP) pathway, can counter dilutional hyponatremia. Furthermore, in acute sodium loading experiments, GC treatment exhibited potent natriuretic effects while suppressing the epithelial sodium channel (ENaC) and AVP-V2 receptor pathway (Zhu X et al., 2020). Given the established association between DCM and HF, this study postulates the potential applicability of glucocorticoids in treating DCM (Walker et al., 2021). Consequently, it is suggested that glucocorticoids hold promise as a therapeutic avenue for DCM.

In this investigation, it was observed that glucocorticoids exhibited a notable capacity to enhance both cardiac and renal functions in individuals with DCM, as well as in rat models of DCM. This effect was accompanied by an upregulation in the expression of NPR1, suggesting a promising avenue for advancing DCM treatment strategies. Moreover, NPR1 emerges as a potentially pivotal target for glucocorticoid therapy in the context of DCM. Nevertheless, it is important to acknowledge certain limitations in our study. The evidence presented here is predominantly derived from a tissue-level analysis. It is imperative that future research incorporates cytological experiments to substantiate our findings. Additionally, current investigations into NPR1 expression in DCM patients have predominantly centered around blood samples. It is our intention to expand our research by including heart tissue from DCM patients, thereby providing further validation for our conclusions. Besides, further clinical and translational studies are required to assess the potential therapeutic applications of glucocorticoids and other interventions targeting NPR1 in patients with DCM, particularly those with diuretic resistance and cardiorenal syndrome. By deepening our knowledge of the underlying mechanisms, we can pave the way for the development of novel treatment strategies to improve the outcomes of patients with DCM.

## Data Availability

The raw data supporting the conclusion of this article will be made available by the authors, without undue reservation.
