# Supplementary figures and images for: Investigating the role of NPR1 in dilated cardiomyopathy and its potential as a therapeutic target for glucocorticoid therapy
Source: Front Pharmacol. 2023 Nov 7;14:1290253. doi: 10.3389/fphar.2023.1290253 (PMC10662320; doi:10.3389/fphar.2023.1290253)

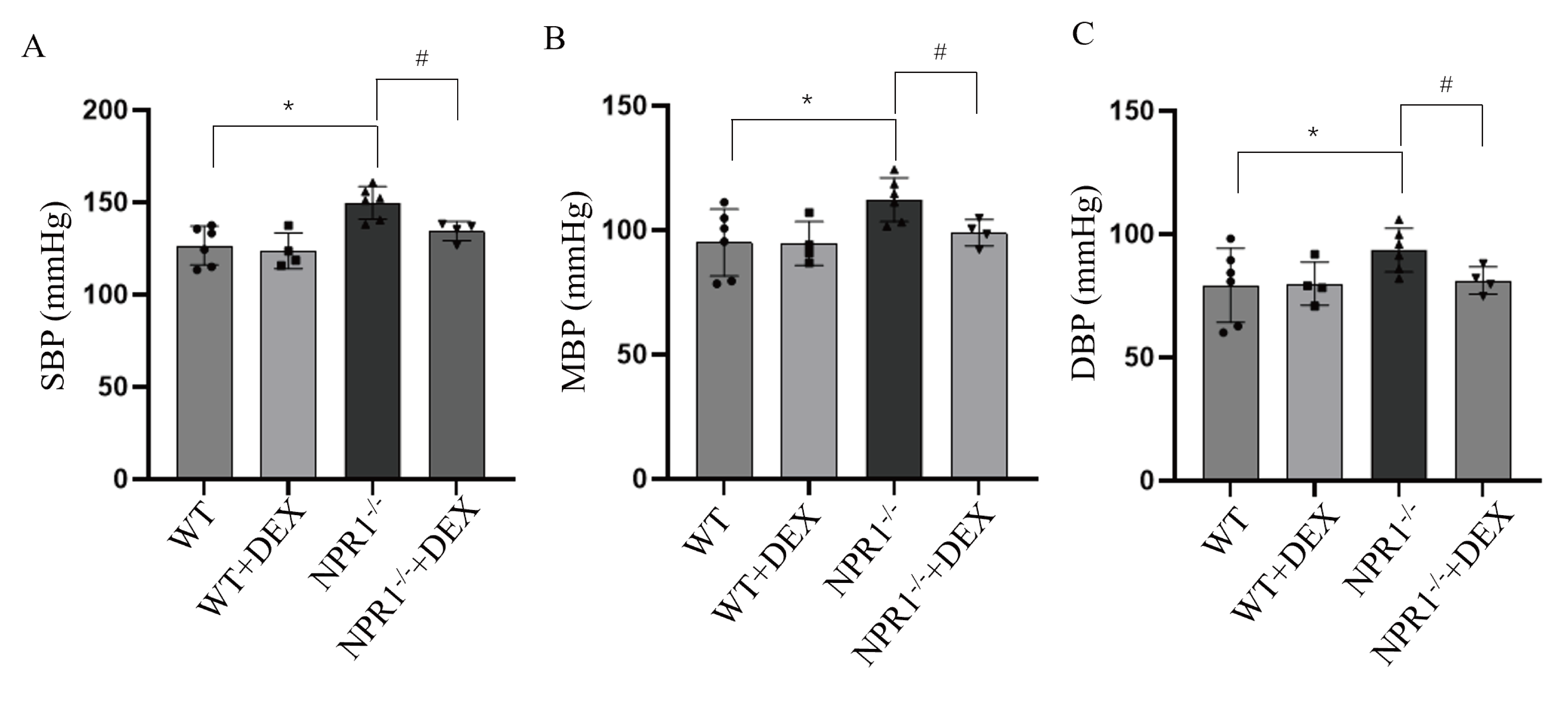

Supplement: Supplementary file 2 [file Image6.TIF]

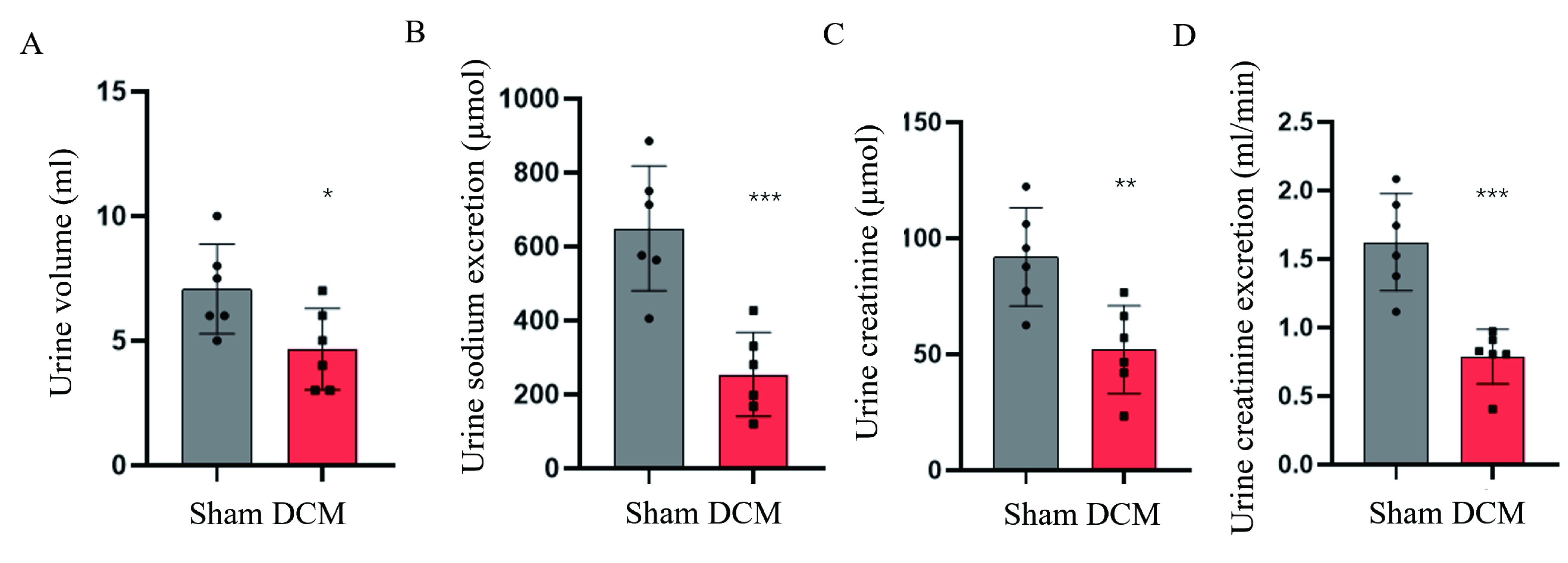

Supplement: Supplementary file 3 [file Image3.TIF]

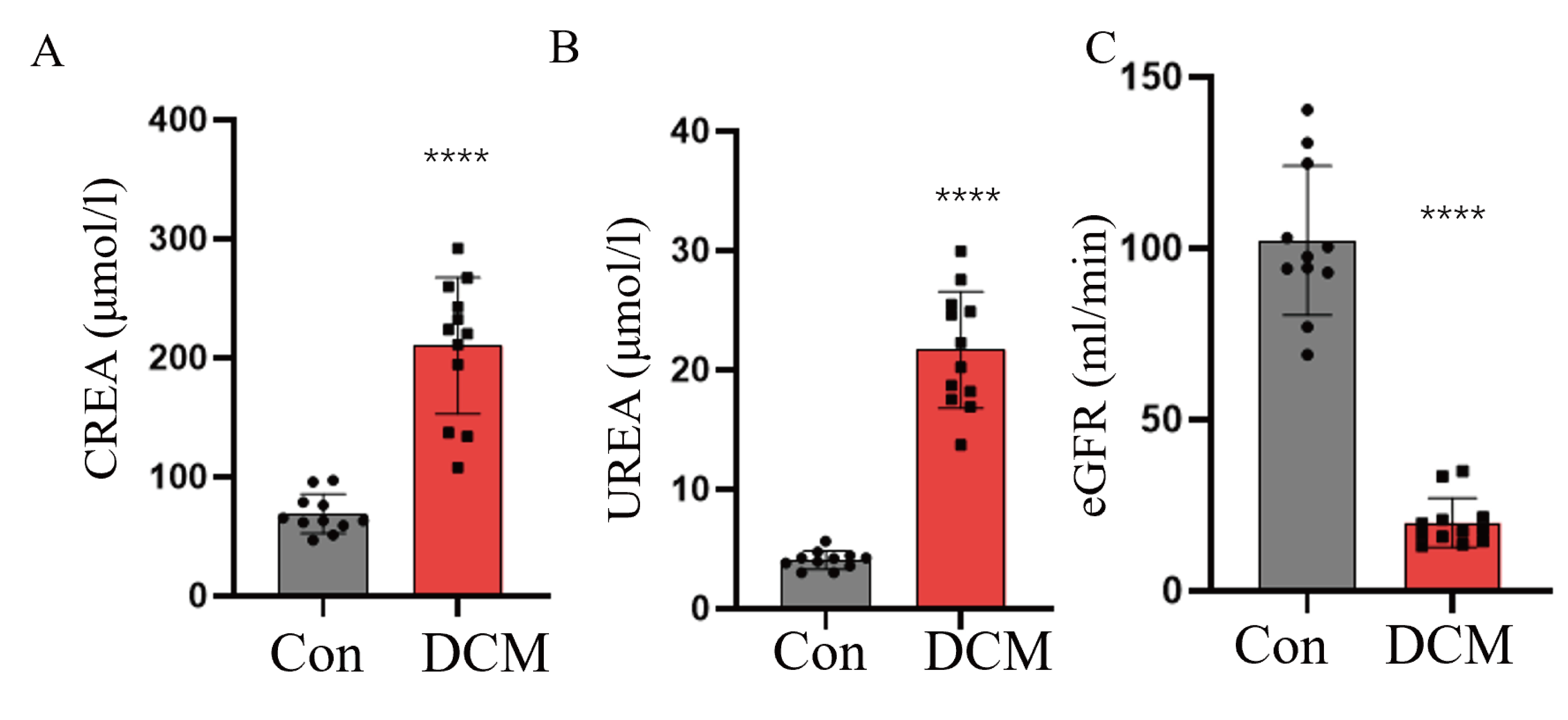

Supplement: Supplementary file 4 [file Image4.tif]

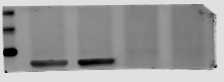

Supplement: Supplementary file 5 [file Image9.TIF]

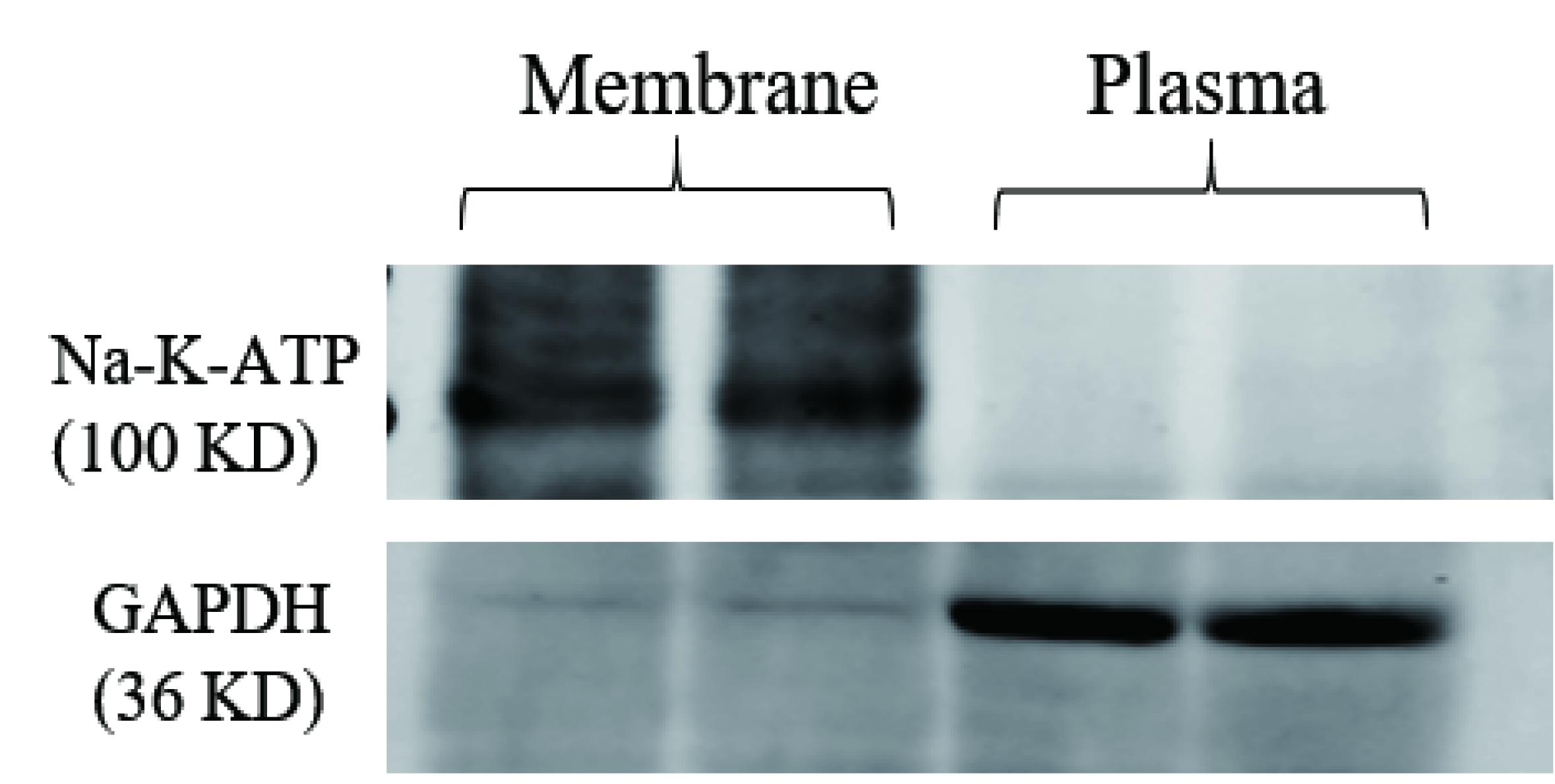

Supplement: Supplementary file 6 [file Image2.TIF]

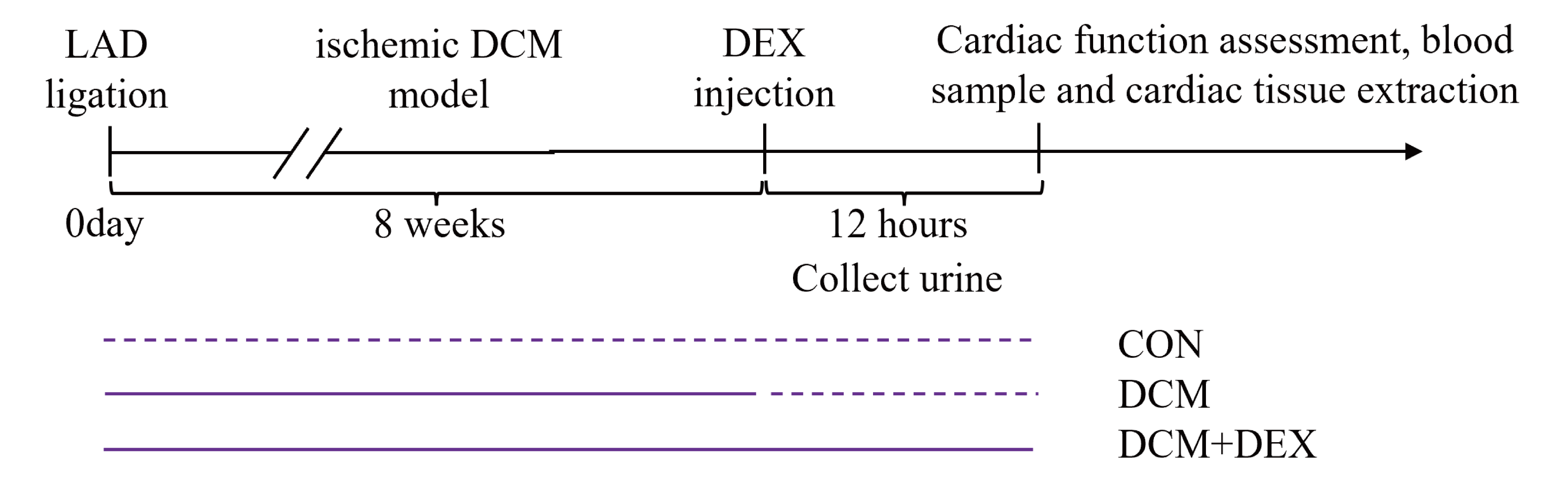

Supplement: Supplementary file 7 [file Image1.TIF]

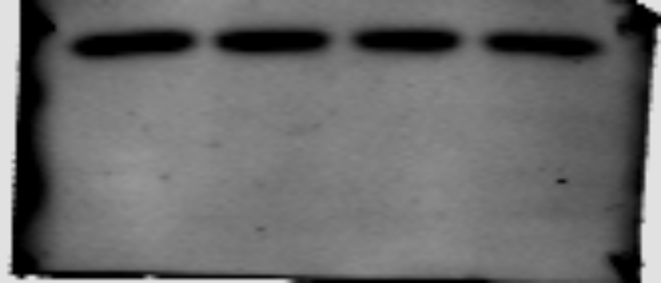

Supplement: Supplementary file 8 [file Image10.TIF]

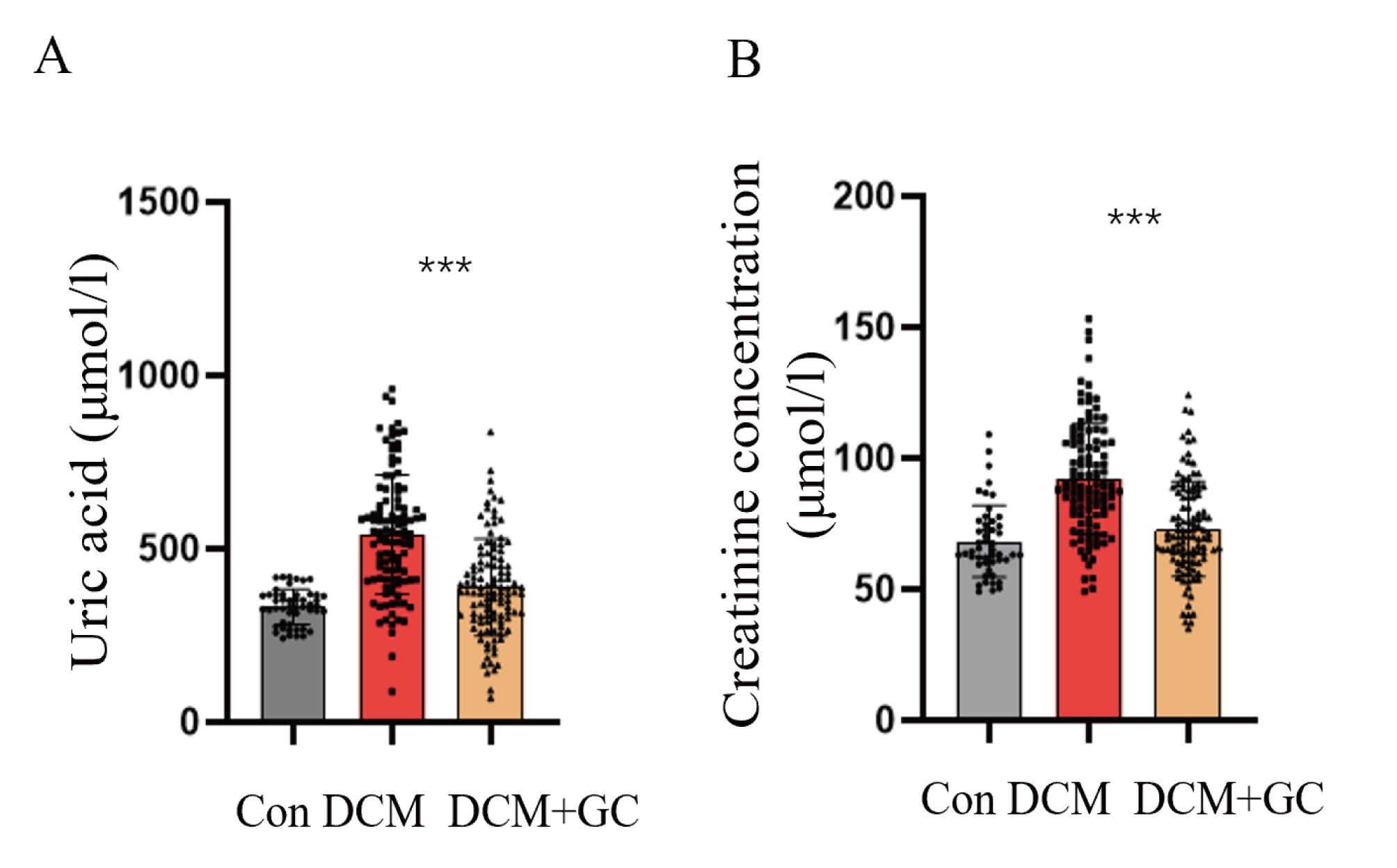

Supplement: Supplementary file 9 [file Image7.tif]

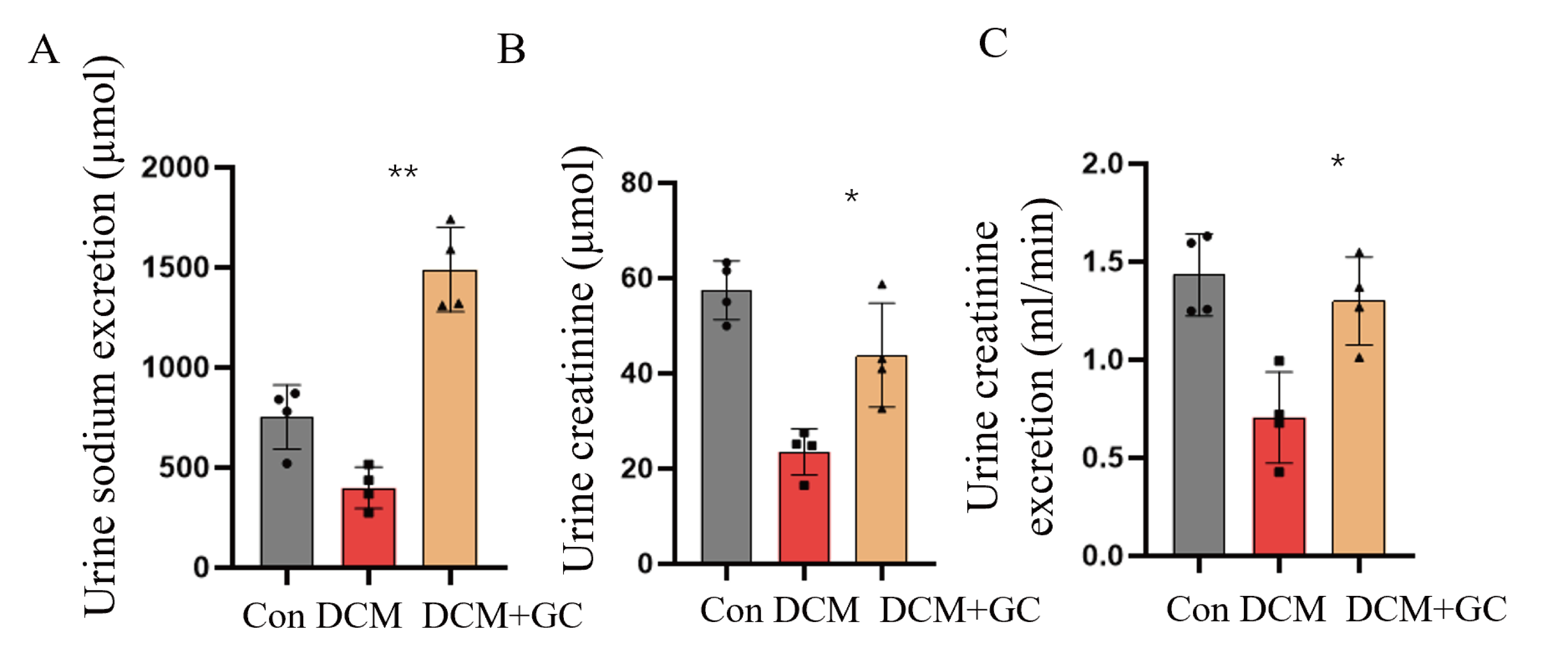

Supplement: Supplementary file 12 [file Image8.tif]

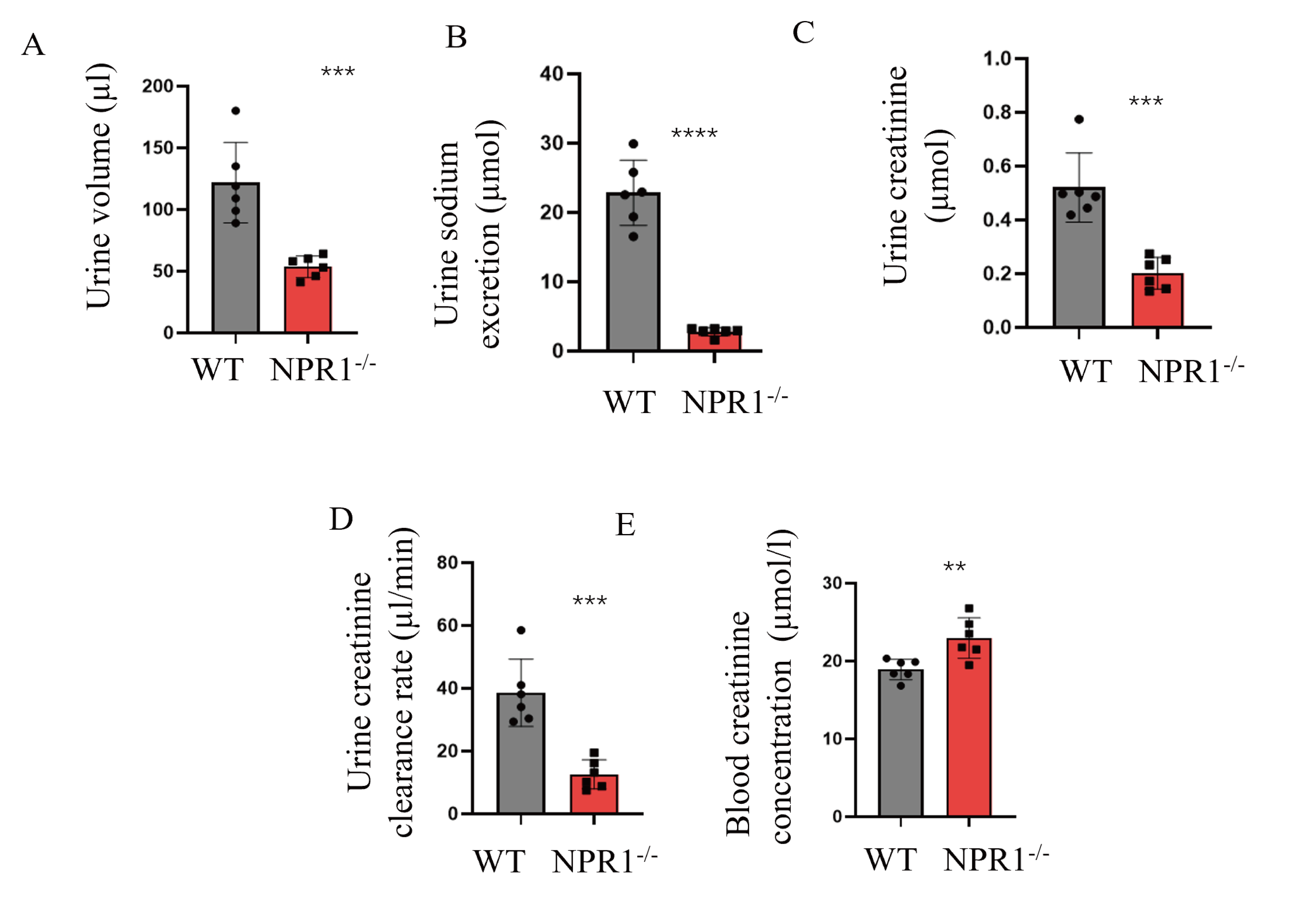

Supplement: Supplementary file 13 [file Image5.tif]
