# Supplementary material for: Investigating the role of NPR1 in dilated cardiomyopathy and its potential as a therapeutic target for glucocorticoid therapy
Source: Front Pharmacol. 2023 Nov 7;14:1290253. doi: 10.3389/fphar.2023.1290253 (PMC10662320; doi:10.3389/fphar.2023.1290253)
Supplement: Supplementary file 10 [file Table2.DOCX]

**The detailed experimental procedure**

Dexamethasone was selected as the representative drug for glucocorticoids in this study. Dexamethasone was administrated 8 weeks after surgery. The rats were randomly divided into three groups (n=8/group): sham surgery, DCM, and DCM+dexamethasone (1 mg/kg, intramuscular injection). The detailed experimental procedure is illustrated in the supplementary figure 1.

**Membrane protein and plasma protein are effectively separated.**

The purity of the extracted membrane is assessed by reacting with GAPDH antibody (Proteintech, Wuhan, China) and Na-K-ATP antibody (Abcam, USA, ab76020). The plasma membrane of extracted myocardial tissue contains almost no GAPDH, and the plasma protein of extracted myocardial tissue contains almost no Na-K-ATP, indicating that our extracted tissue is relatively pure (supplementary figure 2).

**Baseline data in CON and DCM groups.**

There were no statistical differences in Sex, Age, Drinking, Diabetes, Smoking, Renal disease, Cerebral infarction, Cerebral hemorrhage, family history and tumor in Table 1.

**Baseline data in CON, DCM and DCM +DEX groups.**

There was no significant statistical difference in the following aspects, including Sex, Age, Coronary heart disease, Hypertension, Hyperlipidemia, Atrial fibrillation or flutter, Valvular disease, Peripheral vascular disease, PCI, CABG, TIA, COPD, CKD, Thyroid dysfunction, AMI, Abnormal liver function and Anemia in Table 2.

**Renal Function Decreased Significantly in Ischemic DCM Model Rats**

Biochemical results showed that, compared with the sham group, the DCM group exhibited a decrease in urine volume, urinary sodium, urinary creatinine, and creatinine clearance (Supplementary Figure 3A–3D).

**Renal Function Impaired in DCM Patients**

A total of 11 patients with DCM and 12 healthy control subjects (CON group) were included, with no statistical differences in sex, age, smoking status, presence of renal disease, or diabetes (Supplementary Table 1). At this time, the DCM group exhibited a significant increase in blood creatinine and blood urea levels and a dominant decrease in the glomerular filtration rate (GFR) (Supplementary Figure 4A–4C).

**NPR1 KO Impaired Renal Function in Mice**

Biochemical results showed that, compared to WT mice, NPR1^-/-^ mice showed a decrease in urine volume, urinary sodium, urinary creatinine, creatinine clearance and blood creatinine concentration (Supplementary Figure 5A–5E).

**Blood pressure in different groups**

Systolic blood pressure (SBP), diastolic blood pressure (DBP) and mean arterial pressure (MBP) were validated in WT and NPR1 KO mice. The results indicate that compared with WT mice, NPR1 KO mice showed a significant increase in SBP, DBP, and MBP. After DEX treatment, the above indicators showed a significant decrease and tended to normal (Supplementary Figure 6A-6C).

**Glucocorticoids Improved Renal Function in the DCM Group and Upregulated NPR1 mRNA Expression**

There were no statistically significant differences in sex, age, coronary heart disease, hypertension, hyperlipidemia, atrial fibrillation or flutter, valvular disease, peripheral vascular disease, percutaneous coronary intervention (PCI), coronary artery bypass graft surgery (CABG), transient ischemic attack (TIA), chronic obstructive pulmonary disease (COPD), chronic kidney disease (CKD), thyroid dysfunction, acute myocardial infarction (AMI), abnormal liver function, or anemia (Supplementary Table 2). Biochemical results revealed that, compared with the CON group, patients with DCM exhibited a visible increase in urea creatinine, and uric acid levels, which substantially decreased after glucocorticoid administration (Supplementary Figure 7A and 7B).

**Glucocorticoids Improved Renal Function in DCM Rats and Upregulated NPR1 Expression**

Biochemical results showed that compared with the CON group, patients with DCM experienced significant fluctuations in urinary sodium, creatinine, and creatinine clearance, which significantly increased after glucocorticoid administration (Supplementary Figure 8A-8C).

**Supplementary Figure legends**

**Supplementary Figure 1.** detailed experimental procedure.

**Supplementary Figure 2.** The plasma membrane of extracted myocardial tissue contains almost no GAPDH, and the plasma protein of extracted myocardial tissue contains almost no Na-K-ATP, indicating that our extracted tissue is relatively pure.

**Supplementary Figure 3**. Changes in cardiac and renal function indicators in DCM rats. (A–D) urine volume, urinary sodium, urinary creatinine, and creatinine clearance rate in rats (CON, n=6; DCM, n=6). * P<0.05, ** P<0.01, *** P<0.001 vs Sham group. Abbreviations: EF, ejection fraction; FS, fractional shortening; LVESV, left ventricular end-systolic volume; LVESD, left ventricular end-systolic diameter.

**Supplementary Figure 4**. Changes in cardiac and renal function indicators in patients with DCM. (A–C) CREA, UREA, and eGFR in different groups (CON, n=11; DCM, n=12). **** P<0.0001 vs Con group. Abbreviations: LVEDD, left ventricular end-diastolic diameter; EF, ejection fraction; LVEDV, left ventricular end-diastolic volume; LVESV, left ventricular end-systolic volume; FS, fractional shortening; LVESD, left ventricular end-systolic diameter; CREA, creatinine; UREA, urea; eGFR, estimated glomerular filtration rate.

**Supplementary Figure 5**. Change in cardiac and renal function indicators in KO mice. (A–E) urinary volume, sodium, creatinine, creatinine clearance rate, and blood creatinine concentration in different groups of mice (WT n=6, NPR1^-/-^ n=6). * P<0.05, *** P<0.001 vs WT group. Abbreviations: EF, ejection fraction; LVESD, left ventricular end-systolic diameter; LVEDD, left ventricular end-diastolic diameter; LVESV, left ventricular end-systolic volume; LVEDV, left ventricular end-diastolic volume; FS, fractional shortening.

**Supplementary Figure 6**. Change in blood pressure indicators in different groups. (A) SBP indifferent groups; (B) MBP indifferent groups; (C) DBP indifferent groups; Data are presented as mean ± SD (WT n=6, WT+DEX n=4, NPR1^-/-^ n=6, NPR1^-/-^+DEX n=6). * P<0.05 vs Con group. ^#^ P<0.05 vs NPR1^-/-^ group. Abbreviations: SBP, systolic blood pressure; MBP, mean blood pressure; DBP, diastolic blood pressure.

**Supplementary Figure 7.** Glucocorticoids improve cardiac and renal function indicators in patients with DCM. (A–B) Each group showing uric acid and creatinine concentrations in different groups (CON, n=49; DCM, n=110; and DCM+DEX, n=110). * P<0.05, ** P<0.01, *** P<0.001 vs DCM group. Abbreviations: EF, ejection fraction; RVEDD, right ventricular end-diastolic diameter.

**Supplementary Figure 8.** Glucocorticoids improve cardiac and renal function indicators in DCM rats. (A–C) Urinary sodium, urinary creatinine, and creatinine clearance rates varied among different groups. (CON, n=4; DCM, n=4; and DCM+DEX, n=4). * P<0.05, *** P<0.001, *** P<0.0001 vs DCM group. Abbreviations: EF, ejection fraction; FS, fractional shortening; LVESD, left ventricular end-systolic diameter; LVEDV, left ventricular end-diastolic volume; LVEDD, left ventricular end-diastolic Diameter; LVESV, left ventricular end-systolic volume.
